# Polygenic risk for coronary artery disease is associated with cognitive ability in older adults

**DOI:** 10.1093/ije/dyv354

**Published:** 2016-01-28

**Authors:** Saskia P Hagenaars, Sarah E Harris, Toni-Kim Clarke, Lynsey Hall, Michelle Luciano, Ana Maria Fernandez-Pujals, Gail Davies, Caroline Hayward, Generation Scotland, John M Starr, David J Porteous, Andrew M McIntosh, Ian J Deary

**Affiliations:** ^1^ Centre for Cognitive Ageing and Cognitive Epidemiology, University of Edinburgh, Edinburgh, UK; ^2^ Department of Psychology, University of Edinburgh, Edinburgh, UK,; ^3^ Division of Psychiatry, University of Edinburgh, Royal Edinburgh Hospital, Edinburgh, UK,; ^4^ Institute for Genetics and Molecular Medicine, Western General Hospital, University of Edinburgh, Edinburgh, UK and; ^5^ Geriatric Medicine Unit, University of Edinburgh, Royal Infirmary of Edinburgh, Edinburgh, UK

**Keywords:** Coronary artery disease, polygenic traits, cognition, ageing, dementia, genetics

## Abstract

**Background:** Coronary artery disease (CAD) is associated with cognitive decrements and risk of later dementia, but it is not known if shared genetic factors underlie this association. We tested whether polygenic risk for CAD was associated with cognitive ability in community-dwelling cohorts of middle-aged and older adults.

**Methods:** Individuals from Generation Scotland: Scottish Family Health Study (GS:SFHS, *N* = 9865) and from the Lothian Birth Cohorts of 1921 (LBC1921, *N* = 517) and 1936 (LBC1936, *N* = 1005) provided cognitive data and genome-wide genotype data. Polygenic risk profile scores for CAD were calculated for all of the cohorts using the largest available genome-wide association studies (GWAS) data set, the CARDIoGRAM consortium (22 233 cases and 64 762 controls). Polygenic risk profile scores for CAD were then tested for their association with cognitive abilities in the presence and absence of manifest cardiovascular disease.

**Results:** A meta-analysis of all three cohorts showed a negative association between CAD polygenic risk and fluid cognitive ability (β = −0.022, *P* = 0.016), verbal intelligence (β = −0.024, *P* = 0.011) and memory (β = −0.021, *P* = 0.028).

**Conclusions:** Increased polygenic risk for CAD is associated with lower cognitive ability in older adults. Common genetic variants may underlie some of the association between age-related cognitive decrements and the risk for CAD.

Key Messages

- Coronary artery disease is associated with lower cognitive ability.
- This study examines the association between polygenic risk for coronary artery disease and cognitive ability in a large sample ( *N* = 11 387).
- The findings imply a shared genetic architecture between coronary artery disease and lower cognitive ability.
- Identifying associated pathways may provide novel drug targets for these common conditions.

## Introduction

Age-related cognitive decline is an important aspect of health in older people. Accelerated cognitive ageing is associated with greater mortality and morbidity, less independence, lower quality of life and increased dementia risk. ^1^ Since dementia is a substantial and growing burden on ageing populations, ^2^ it is important to understand the mechanisms underpinning cognitive ability and ageing.

Some cognitive abilities decline on average as people grow older, including aspects of memory, speed of thinking and abstract reasoning. ^1^ Age-related cognitive decline shows substantial variation in the population ^3^^,^^4^ and this has spurred attempts to discover the factors influencing cognitive abilities in later life. Genetic and environmental risk factors both make a substantial contribution to people’s differences in their level of cognitive ability. Twin studies suggest that the heritability of cognitive ability is above 50% in adulthood, including in older age. ^5–7^ Genome-wide association studies (GWAS) suggest that around one-third of the variation in cognitive ability is accounted for by common genetic variants. ^8^^,^^9^

A proportion of the variation in cognitive ability levels is probably due to disease states. Coronary artery disease (CAD) is one type of cardiovascular disease (CVD) robustly associated with reduced cognitive ability. ^10–15^ It tends to be assumed that the direction of the causation between CAD and cognitive ability is that more CAD leads to lower cognitive ability. There is considerable evidence indicating the reverse direction also, whereby lower cognitive ability in childhood represents a risk factor for CAD and is associated with higher morbidity and mortality for CAD. ^16^^,^^17^ Whereas there is a substantial environmental contribution to CAD, family and twin studies suggest that CAD is also substantially heritable, with approximately 40% to 50% of its susceptibility being accounted for by genetic factors. ^18^ Based on a heritability of 40%, recent GWAS studies have shown that 45 confirmed CAD susceptibility loci explained 6% of the additive genetic variance for CAD, which increased to 10.6% after the addition of 104 nominally associated variants. ^19^

In summary, CAD and cognition are correlated phenotypically, and population variation in each is caused by genetic and environmental factors. Luciano *et al.*^20^ showed that some associations between risk factors for CVD and cognitive ability are explained by genetic factors. Therefore, it is possible that the association between CAD and cognitive ability may in part be due to shared genetic variation; that is, some genetic risk factors might confer risk of both CAD and lower cognitive ability level. In order to test whether there is a genetic correlation between CAD and cognitive ability, cohorts consisting of cognitively healthy individuals with both genome-wide genotyping and cognitive data are required.

Because of their phenotypic correlation and the heritable and polygenic nature of both CAD and cognitive ability, we hypothesized that these traits have shared genetic causes. We tested the hypothesis using a polygenic risk profiling approach, ^21^ using the Lothian Birth Cohorts (LBCs) and the Generation Scotland: Scottish Family Health Study Cohort (GS:SFHS) in the presence and absence of manifest CVD.

## Methods

### Cohorts and cognitive measures

#### GS:SFHS

GS:SFHS is a recently available population-based cohort of over 21 000 people. ^22^^,^^23^ Genome-wide single nucleotide polymorphism (SNP) data were ascertained for 9865 individuals, 5790 female and 4075 male, with a mean (standard deviation; SD) age of 52.2 (13.6) years. Details of GS:SFHS are provided in the Supplementary material (available as Supplementary data at *IJE* online) and the derived cognitive measures are shown in Table 1 . In short, the cohort included measures of verbal intelligence ( *N* = 9697), memory ( *N* = 9748), verbal fluency ( *N* = 9753) and processing speed ( *N* = 9732). A derived measure of fluid cognitive ability ( *N* = 9630) was obtained through a principal component analysis (PCA) of the measures for memory, verbal fluency and processing speed, and extracting the first unrotated principal component.

**Table 1.** Cognitive abilities derived from the cognitive test battery of GS:SFHS, LBC1921 and LBC1936

	GS:SFHS	LBC1921	LBC1936
Fluid cognitive ability	Logical Memory ^†^ Verbal Fluency * Digit Symbol-Coding *	Moray House Test Raven’s Standard Progressive Matrices ^‡^ Total Logical Memory ^‡^ Verbal Fluency	Digit Span Backwards ^†^ Matrix Reasoning * Letter-Number Sequencing * Block Design * Symbol Search * Digit Symbol *
Verbal intelligence	Mill Hill Vocabulary Test	National Adult Reading Test	National Adult Reading Test
Memory	Logical Memory ^†^	Total Logical Memory ^‡^	Logical Memory I Total Recall ^†^ Logical Memory Ii Delayed Total Recall ^†^ Spatial Span Forward ^†^ Spatial Span Backward ^†^ Verbal Paired Associates I & II ^†^ Letter-Number Sequencing * Digit Span Backwards *
Processing speed	Digit Symbol-Coding *	Digit Symbol * 4-Choice Reaction Time mean Inspection Time mean	Digit Symbol * Choice Reaction Time mean Simple Reaction Time mean Inspection Time Symbol Search

Test references are shown in the supplemental methods .

^†^ Wechsler Memory Scale-III.

*Wechsler Adult Intelligence Scale-III.

^‡^ Wechsler Memory Scale-Revised.

#### LBC1921 and LBC1936

The LBCs of 1921 and 1936 are longitudinal studies providing lifelong cognitive data between the ages of 11 and 79, and genome-wide SNP data for a total of 1522 older individuals (LBC1921 = 517 individuals, 302 female and 215 male, LBC1936 = 1005 individuals, 496 female and 509 male). ^24^^,^^25^ The Lothian Birth Cohorts were originally identified through contacting individuals living in the Edinburgh area of Scotland (Lothian) who were born in 1921 or 1936 and might therefore have taken part in the Scottish Mental Surveys (SMS) at age about 11 years in 1932 or 1947, respectively. In these surveys, almost every child born in those years and attending school in Scotland completed the Moray House Test No. 12 (MHT) assessment of general intelligence. All participants of both LBCs completed an additional cognitive test battery around the ages of 79 and 83 in LBC1921 and age 70 in LBC1936, including a measure of verbal intelligence (total *N* = 1515). Derived measures of memory (total *N* = 1477), processing speed (total *N* = 1253) and fluid cognitive ability (total *N* = 1491) were obtained through a PCA and extracting the first unrotated principal component. Details of the LBCs and derived cognitive measures are shown in the Supplementary material (available as Supplementary data at *IJE* online) and in Table 1 .

### Genotyping

DNA extracted from venous blood from the participants was genotyped at the Wellcome Trust Clinical Research Facility using the Illumina HumanOmniExpressExome-8 v1.0 DNA Analysis Beadchip and Infinium chemistry (GS:SFHS), ^26^^,^^27^ and the Illumina 610-Quadv1 whole-genome SNP array (LBC’s). The sample collection, quality control and genotyping process is described elsewhere in more detail. ^8^^,^^23^ Four (five for GS:SFHS) MDS components were calculated and extracted using the SNP data set to control for population stratification. Scree plots of the first 10 MDS components for population stratification in GS:SFHS and the first four in both LBCs are shown in Supplementary Figure 1a–c (available as Supplementary data at *IJE* online).

### Creating CAD polygenic risk scores

The method to create polygenic risk scores (PGRS) has been described previously. ^21^ In summary, this method calculates SNP effect sizes from published genetic association data and calculates the genome-wide weighted sum of the alleles that an individual carries. This sum, the polygenic risk score, then serves as an index of the genetic load for a specific disease. Summary statistics from the CARDIoGRAM consortium (22 233 cases and 64 762 controls) were used to create the CAD PGRS. Details of the methods used by CARDIoGRAM can be found elsewhere. ^28^ The training sample data results for each SNP were thresholded at cut-offs of 0.01, 0.05, 0.1, 0.5 and 1. After linkage disequilibrium pruning (based on *r ^2^* > 0.25 within a 200-SNP sliding window), the five resulting SNP sets were used to construct a risk profile for the subjects in GS:SFHS, LBC1921 and LBC1936, using the cumulative sum of each SNP allele dose multiplied by the log of the odds ratio derived from CARDIoGRAM. Raw genotype data were used to create the risk scores for both LBCs and GS:SFHS. The number of SNPs included in each threshold in the three cohorts can be found in Supplementary Table 1 (available as Supplementary data at *IJE* online). Throughout the paper, only the data using the cut-off of 0.5 will be reported as this threshold has shown to be the most predictive threshold in a large independent sample (UK Biobank, *n* = 112 151, Supplementary Table 2 , available as Supplementary data at *IJE* online).

### Statistical analysis

All statistical analyses were conducted in the R statistical software package. ^29^ Point-biserial correlation coefficients were calculated between self-reported CVD and the cognitive phenotypes. At all five SNP thresholds, linear regression models were created between CAD PGRS and self-reported CVD, as proof of principle, and the cognitive phenotypes, adjusting for the four MDS components for population stratification, age and sex in both LBC1921 and LBC1936.

The GS:SFHS models were also adjusted for family structure by fitting a univariate linear mixed model which estimates the genetic and environmental variance, using the ASReml program. ^30^ The inverse of a relationship matrix was created by using pedigree kinship information to fit the family structure as a random effect. The cognitive scores were used as the dependent variable and age, sex, the five MDS components and the PGRS were used as fixed effects. Wald’s conditional F-test was used to calculate *P* -values for the fixed effects. Additional analyses were performed adjusting the fluid cognitive ability models for verbal intelligence and the verbal intelligence models for fluid cognitive ability.

An analysis by age group (<40 years, 40–60 years, >60 years) in GS:SFHS was performed to test if CAD PGRS had different associations with cognition in different age groups. An analysis excluding participants with self-reported CVD was performed to test if any associations with cognitive ability were being driven by participants with CVD.

A meta-analysis, using the meta package, of the results across studies was then conducted in order to synthesize the findings for maximum statistical power and to check for heterogeneity. A fixed-effects model was used in which the standardized regression coefficients were weighted by the inverse of their squared standard error and pooled to provide a summary estimate across both cohorts, based on the method of the DerSimonian-Laird estimator. ^31^ We tested for the presence and magnitude of between-study heterogeneity using Cochran’s Q and the I ^2^ statistic, respectively. ^32^

To test whether different CAD SNPs are contributing to different cognitive traits, a linear regression was conducted between 25 (of 45) CAD SNPs that were available in the largest data set (GS:SFHS), adjusting for age, sex and five MDS components for population stratification.

## Results

A positive CVD history was observed in 30.2 % of LBC1921 ( *n* = 156), 24.6 % of LBC1936 ( *n* = 247) and 4.7 % of GS:SFHS ( *n* = 471). All cognitive traits were approximately normally distributed, with outliers > ± 3.5 SDs from the mean excluded. There were many (9 of 15) small point-biserial correlations between self-reported history of CVD and lower scores for the cognitive phenotypes in the larger GS:SFHS and LBC1936 cohorts, but not in LBC1921, though the latter had similar effect sizes ( Table 2 ). Phenotypic correlations between the different cognitive ability traits in each cohort can be found in Supplementary Table 3a–c (available as Supplementary data at *IJE* online). Correlations between the polygenic risk scores in each cohort can be found in Supplementary Table 4a–c (available as Supplementary data at *IJE* online).

**Table 2.** Phenotypic correlations (point-biserial) for self-reported cardiovascular disease and each of the cognitive traits for the three cohorts

	Self-reported cardiovascular disease		
	GS:SFHS	LBC1921	LBC1936
VI	0.01	−0.08	−0.07 *
Memory	−0.07**	−0.07	−0.10**
PS	−0.16**	−0.14 *	−0.14**
VF	−0.02 *	−0.08	−0.05
G _fluid_	−0.12**	−0.05	−0.13**

VI, verbal intelligence; PS, processing speed; VF, Verbal Fluency test; G _fluid_ , fluid cognitive ability.

* *P* < 0.05; ** *P* < 0.005.

**Figure 1.**
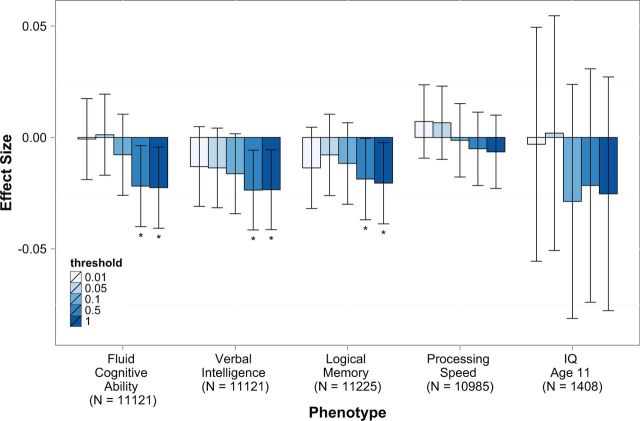
Meta-analysis of associations between coronary artery disease polygenic risk and cognitive traits at five SNP thresholds. Plot shows estimates with 95% confidence intervals; results with *P* -values below 0.05 are indicated by an asterisk.

A fixed-effects meta-analysis of the cognitive phenotypes in all three cohorts showed that CAD polygenic risk was positively associated with self-reported CVD at all SNP inclusion thresholds ( Table 3 , Figure 1 ). CAD polygenic risk was associated with fluid cognitive ability (β = −0.0213, *P* = 0.0195), with verbal intelligence (β = −0.0238, *P* = 0.0107) and with memory (β = −0.0186, *P* = 0.0461). No heterogeneity was reported except for CVD history at a threshold of *P* < 0.01 (I ^2^ = 72.7%, *P* = 0.03) ( Supplementary Table 5 , available as Supplementary data at *IJE* online).

**Table 3.** Effect size and significance of meta-analysis correlations between CAD polygenic risk scores and cognitive abilities or self-reported history of cardiovascular disease

CAD genetic risk scores: SNPs with *P* -values <		Fluid cognitive ability	Verbal intelligence	Memory	Processing speed	IQ age 11	CVD history
		*n* = 11121	*n* = 11121	*n* = 11225	*n* = 10985	*n* = 1408	*n* = 7930
1	β	−0.0220	−0.0237	−0.0205	−0.0063	−0.0253	0.1806
	z	−2.41	−2.53	−2.19	−0.74	−0.081	4.15
	*P* -value	**0.0161**	**0.0113**	**0.0284**	0.4567	0.3444	**3.33 × 10** ^−^ **^5^**
0.5	β	−0.0213	−0.0238	−0.0186	−0.0049	−0.0216	0.1737
	z	−2.34	−2.55	−1.99	−0.59	−0.807	4.00
	*P* -value	**0.0195**	**0.0107**	**0.0461**	0.559	0.4197	**6.40 × 10** ^−^ **^5^**
0.1	β	−0.0072	−0.0160	−0.0114	−0.0007	−0.0287	0.1818
	z	−0.79	−1.71	−1.22	−0.09	−1.07	4.20
	*P* -value	0.429	0.0865	0.2207	0.9313	0.2843	**2.70 × 10** ^−^ **^5^**
0.05	β	0.0014	−0.0135	−0.0077	0.0070	0.0019	0.1341
	z	0.16	−1.45	−0.82	0.83	0.07	3.12
	*P* -value	0.8759	0.1477	0.411	0.406	0.9429	**0.0018**
0.01	β	−0.0003	−0.0128	−0.0135	0.0078	−0.003	0.1006
	z	−0.03	−1.38	−1.44	0.92	−0.1137	2.33
	*P* -value	0.9742	0.1716	0.1489	0.3571	0.9095	**0.0194**

Effects with *P* < 0.05 are shown in bold.

CAD, coronary artery disease; CVD history, history of cardiovascular disease; β, standardized regression coefficient.

Individual analysis of the three cohorts showed that CVD status was associated with CAD PGRS in GS:SFHS (β = 0.202, *P* = 0.0013) and LBC1921 (β = 0.231, *P* = 0.0225). No association was found between CVD status and CAD PGRS in LBC1936 and LBC1921 ( Supplementary Tables 6 and Supplementary Data , available as Supplementary data at *IJE* online).

In GS:SFHS, CAD PGRS showed an association with the Mill Hill Vocabulary (MHV) test (β = −0.03, *P* = 0.004), with Memory (β = −0.02, *P =* 0.035) and with fluid cognitive ability (β = −0.02, *P* = 0.020). No associations were found for the other cognitive phenotypes. The direction of effect for all associations of the cognitive phenotypes with CAD PGRS was negative and occurred in the proposed direction of the hypothesis: greater CAD PGRS is associated with lower cognitive test scores. A subset without individuals with self-reported CVD showed comparable results ( Supplementary Table 6 ). No associations were found for the age group below 40 years ( *n* = 1831), or the group between 40 and 60 years ( *n* = 5204). In the age group above 60 years ( *n* = 2825) CAD PGRS showed an association with fluid cognitive ability (β = −0.037, *P* = 0.044), as well as verbal fluency (VF) (β = −0.063, *P* = 0.001) ( SupplementaryTable 7 , available as Supplementary data at *IJE* online).

Additional analyses adjusting the verbal intelligence models for fluid cognitive ability showed that verbal intelligence remains associated with CAD polygenic risk (β = −0.009, *P* = 0.29). Fluid cognitive ability is not associated with CAD polygenic risk after adjusting for verbal intelligence (β = −0.02, *P* = 0.03). No associations were found between CAD PGRS and the cognitive phenotypes in LBC1921 and LBC1936 ( Supplementary Table 8 , available as Supplementary data at *IJE* online).

In GS:SFHS, no associations were found between 25 SNPs, which passed a threshold for association for CAD, and the cognitive traits ( Supplementary Table 9 , available as Supplementary data at *IJE* online).

## Discussion

Based on SNP associations from a large GWAS study of CAD, PGRS were created for CAD in the three independent cohorts measuring cognition in middle to old age. CAD PGRS was associated with CVD history at all SNP thresholds in a meta-analysis of all three cohorts. This study found widespread phenotypic associations between CVD history and the cognitive phenotypes in GS:SFHS and LBC1936; people with CVD tended to have lower cognitive ability. In the smaller LBC1921, most associations showed similar-sized correlation coefficients. This study found a negative association between common genetic variants for CAD and fluid cognitive ability in GS:SFHS and in a meta-analysis of all three cohorts. Processing speed and memory were the only traits showing an association in LBC1921, the smallest of the cohorts. No associations were found in LBC1936, though the effects were in the expected direction and comparable to the effects in GS:SFHS. CAD PGRS were also negatively correlated with measures of vocabulary and logical memory in GS:SFHS.

Cognitive ability tends to be lower in individuals suffering from CAD, ^10^^,^^33^ which is supported by our results showing a negative correlation between CVD history and the cognitive phenotypes, and more recent studies have demonstrated that a decline in cognitive abilities may be seen in individuals at high risk for CAD. ^34^ The results of this DNA-based study suggest that the phenotypic association between CAD and cognitive ability has some shared genetic aetiologies. This is supported by the family-based genetic analysis results of Luciano *et al.*^20^ on GS:SFHS.

The GS:SFHS analysis by age group showed negative associations in the age group above 60 years between CAD PGRS and MHV, VF and fluid cognitive ability. This suggests that the decline in cognitive abilities happens when individuals reach the age where they are more at risk for CAD. This is supported by results from the American Heart Association, who showed that the prevalence of CAD doubles in older individuals. ^35^ When fitting an interaction term for age and CAD polygenic risk, we only found an interaction for verbal fluency.

Although the direction of causation tends to be assumed to be from more CAD to less cognitive capability, there is evidence here and elsewhere to lead us to consider the reverse association too. It is now widely replicated that lower general cognitive ability from childhood and young adulthood predicts more morbidity and mortality from CVD in later life. ^17^^,^^36–39^ It is notable that in the present meta-analysis, verbal intelligence was associated with CAD PGRS. This type of vocabulary test tends to indicate peak previous cognitive ability rather than being an indicator of cognitive ageing. Therefore, it is possible that this result represents some genetic confounding such that some of the same genes result in lower vocabulary scores and more CAD. However, we did not find an association for IQ at age 11 with CAD PGRS. It is also conceivable that the CAD PGRS is in part picking up some of the genetic contribution to lifetime-stable cognitive differences and that such cognitive differences are associated with creating lifestyles and environments that are more or less conducive to CAD, as we discuss elsewhere. ^16^^,^^40^

Fluid cognitive ability and verbal intelligence are moderately correlated with each other (r = 0.34). The additional analyses adjusting both verbal intelligence and fluid cognitive ability for each other shows greater attenuation for fluid cognitive ability after controlling for verbal intelligence than the reverse. This could be because verbal intelligence is psychometrically a more reliable measure. Also, these results could indicate that the association with CAD is more due to the stable trait of cognitive ability, typically well assessed by vocabulary measures, rather than to any cognitive decline that might be captured by the more age- and illness-sensitive fluid cognitive ability.

Among the study’s strengths were the large sample size of GS:SFHS, the detailed cognitive testing of different domains in the cohorts, the geographical homogeneity of the three cohorts, the access to the results of a large meta-analysis of GWAS studies of CAD, and the rare ability to examine the association of CAD with cognitive ability data in childhood and older age in both LBCs.

The present study has some limitations. The use of self-reported CVD may have led to misclassification of CVD conditions, causing a likely bias toward the null hypothesis. ^33^ This study is unable to test for a direction of causation between CAD and cognitive ability. Raw *P* -values are presented for the PGRS associations. We acknowledge that multiple tests were performed, but it is difficult to determine the appropriate test to correct for multiple testing because both the traits as the polygenic risk scores are highly correlated ( Supplementary Tables 3a–c and Supplementary Data , available as Supplementary data at *IJE* online).

This study failed to replicate the associations found in GS:SFHS between CAD PGRS and cognition in both LBCs. The sample size of LBC1921 is substantially smaller than the sample sizes of GS:SFHS and LBC1936; therefore LBC1921 probably does not have enough power to detect differences. Together with the higher mean age and the greater comorbidity, this might explain the differences in results. An explanation for the absence of associations in LBC1936 might be the lack of an association between self-reported CVD and CAD PGRS. Nevertheless, the meta-analysis of the associations in all three cohorts supports the overall findings and conclusion.

The present study suggests that CAD genetic risk is negatively associated with cognitive ability in healthy population- based cohorts. These findings were made in general population cohorts and were independent of CAD pathology. These findings suggest that CAD and cognitive ability share some common genetic aetiology. Further annotation of the shared genetic architecture and its associated biological pathways may provide novel drug targets for both disorders.

## Funding

This work was supported by Wellcome Trust Strategic Award [104036/Z/14/Z]. Generation Scotland has received core funding from the Chief Scientist Office of the Scottish Government Health Directorates [CZD/16/6] and the Scottish Funding Council [HR03006]. The research was supported by a programme grant from Age UK (Disconnected Mind) and by grants from the Biotechnology and Biological Sciences Research Council (BBSRC). The work was undertaken by the University of Edinburgh Centre for Cognitive Ageing and Cognitive Epidemiology, part of the cross-council Lifelong Health and Wellbeing Initiative [MR/K026992/1]. Funding from the Medical Research Council (MRC) and BBSRC is gratefully acknowledged.

## Supplementary Material

Supplementary Data
