# Supplementary material for: Polygenic risk for coronary artery disease is associated with cognitive ability in older adults
Source: Int J Epidemiol. 2016 Jan 28;45(2):433–40. doi: 10.1093/ije/dyv354 (PMC4864876; doi:10.1093/ije/dyv354)
Supplement: Supplementary Data [file dyv354_supplementary_data.zip › ije-2015-02-0236-File006.docx]

**Supplemental Methods**

**Cohorts and measures**

**GS:SFHS**

GS:SFHS is a recently available population-based cohort of over 21,000 people. Genome-wide SNP data were ascertained for 9865 individuals, 5790 female and 4075 male, with a mean (SD) age of 52.2 (13.6) years. The study protocol for GS has been described elsewhere^1, 2^. All participants in GS:SFHS were invited to complete a short battery of well-validated cognitive tests, personal and family medical history details were taken during a clinical interview. The four cognitive tests were the Mill Hill Vocabulary test (combined Junior and Senior synonyms)^3^, Logical Memory in the Wechsler Memory Scale III (WMS-III, the sum of immediate and delayed recall for one paragraph)^4^, the phonemic Verbal Fluency test (using letters C, F, and L)^5^ and Digit Symbol-Coding from the Wechsler Adult Intelligence Scale III (WAIS-III)^6^. Fluid cognitive ability was constructed by performing a principal component analysis (PCA) of Logical Memory, Verbal Fluency and Digit Symbol-Coding and extracting the first unrotated principal component.

**LBC1921 and LBC1936**

The Lothian Birth Cohorts of 1921 and 1936 are longitudinal studies and provide lifelong cognitive data between the ages of 11 and 79, and genome-wide SNP data. In these Surveys, almost every child born in those years and attending school in Scotland completed the Moray House Test No. 12 (MHT) assessment of general intelligence. Genome-wide SNP data were ascertained a total of 1522 older individuals (LBC1921 = 517 individuals, 302 female and 215 male , LBC1936 = 1005 individuals, 496 female and 509 male), born in 1921 or 1936^7, 8^. Disease history was obtained during a structured interview.

The MHT test scores were converted to an IQ-type scale with a mean of 100 and SD = 15 for each sample, as described elsewhere^9^. The following cognitive abilities were derived from the cognitive test battery: fluid cognitive ability, verbal intelligence, memory, processing speed. Fluid cognitive ability, the first unrotated component of a principal component analysis (PCA), was derived for LBC1921 from the following tests as described previously^10^: Moray House Test, Raven’s Standard Progressive Matrices, total Logical Memory from the Wechsler Memory Scale-Revised (WMS-R)^11^ and Verbal Fluency. For LBC1936 the tests were: WMS-III Digit Span Backwards and WAIS-III Matrix Reasoning, Letter-Number Sequencing, Block Design, Symbol Search and Digit Symbol. Verbal intelligence was assessed by the National Adult Reading Test (NART) in both the Lothian Birth Cohorts ^12^. Memory was assessed using the total Logical Memory score from the WMS-R in LBC1921. In LBC1936, memory was derived via PCA using WMS-III Logical Memory I total recall score, Logical Memory II Delayed total recall score, Spatial Span Forward, Spatial Span Backward, Verbal Paired Associates I, Verbal Paired Associates II recall total score, and WAIS-III Letter-Number Sequencing and Digit Span Backwards. Processing speed was derived via PCA using Digit symbol, 4-Choice Reaction Time mean and Inspection Time mean in LBC1921 at age 83 years^13^; Choice Reaction Time Mean, Simple Reaction Time mean, Digit Symbol, Inspection Time and Symbol Search in LBC1936^14, 15^. The change in IQ between childhood and old age in the LBC’s was calculated using a linear regression model that summarizes the relation between IQ at age 11 and age 70 (LBC1936) or age 79 (LBC1921). In this model, the residual values reflect the observed deviation of IQ at age 70 or 79 based on IQ predicted at age 11, representing an estimate for change in IQ.

Reference list

1. Smith BH, Campbell H, Blackwood D, et al. Generation Scotland: the Scottish Family Health Study; a new resource for researching genes and heritability. *BMC medical genetics* 2006; **7**: 74.

2. Smith BH, Campbell A, Linksted P, et al. Cohort profile: Generation Scotland: Scottish Family Health Study (GS: SFHS). The study, its participants and their potential for genetic research on health and illness. *International journal of epidemiology* 2012: dys084.

3. Raven J. Court JH, Raven J. *Manual for Raven’s progressive matrices and vocabulary scales* 1977.

4. Wechsler D. *Wechsler Memory Scale (WMS-III)*: Psychological corporation; 1997.

5. Lezak MD. *Neuropsychological assessment*: Oxford university press; 2004.

6. Wechsler D. *WAIS-III: Administration and scoring manual: Wechsler adult intelligence scale*: Psychological Corporation; 1997.

7. Deary IJ, Gow AJ, Pattie A, Starr JM. Cohort profile: the Lothian Birth Cohorts of 1921 and 1936. *International journal of epidemiology* 2012; **41**: 1576-84.

8. Deary IJ, Gow AJ, Taylor MD, et al. The Lothian Birth Cohort 1936: a study to examine influences on cognitive ageing from age 11 to age 70 and beyond. *BMC geriatrics* 2007; **7**: 28.

9. Gow AJ, Corley J, Starr JM, Deary IJ. Reverse causation in activity-cognitive ability associations: the Lothian Birth Cohort 1936. *Psychology and aging* 2012; **27**: 250.

10. Davies G, Tenesa A, Payton A, et al. Genome-wide association studies establish that human intelligence is highly heritable and polygenic. *Molecular psychiatry* 2011; **16**: 996-1005.

11. Wechsler D. *WMS-R: Wechsler Memory Scale-Revised: Manual*: Psychological Corporation San Antonio; 1987.

12. Nelson HE, Willison J. *National Adult Reading Test (NART)*: Nfer-Nelson; 1991.

13. Luciano M, Hansell NK, Lahti J, et al. Whole genome association scan for genetic polymorphisms influencing information processing speed. *Biological psychology* 2011; **86**: 193-202.

14. Luciano M, Mõttus R, Harris S, et al. Predicting cognitive ability in ageing cohorts using Type 2 diabetes genetic risk. *Diabetic Medicine* 2014; **31**: 714-20.

15. Harris SE, Davies G, Luciano M, et al. Polygenic Risk for Alzheimer's Disease is not Associated with Cognitive Ability or Cognitive Aging in Non-Demented Older People. *Journal of Alzheimer's Disease* 2014; **39**: 565-74.
