# Supplementary material for: Polygenic risk for coronary artery disease is associated with cognitive ability in older adults
Source: Int J Epidemiol. 2016 Jan 28;45(2):433–40. doi: 10.1093/ije/dyv354 (PMC4864876; doi:10.1093/ije/dyv354)
Supplement: Supplementary Data [file dyv354_supplementary_data.zip › ije-2015-02-0236-File007.docx]

**Supplementary Materials for:**

Hagenaars et al. Polygenic Risk for Coronary Artery Disease is Associated with Cognitive Ability in Older Adults

**Contents**

**Page 2:** Supplementary Figures 1a-c, on screeplots for principal components in all three cohorts.

**Page 4:** Supplementary Table 1, on the number of SNPs included at each threshold for the polygenic profile scores.

**Page 5:** Supplementary Table 2, on explained variance for cardiovascular disease history for each threshold in UK Biobank.

**Page 6:** Supplementary Table 3a-c, on phenotypic correlations between cognitive variables in all three cohorts.

**Page 7:** Supplementary Table 4a-c, on correlations between polygenic risk scores in all three cohorts.

**Page 8:** Supplementary Table 5, on between study heterogeneity.

**Page 9:** Supplementary Table 6, on the full analyses between cognitive traits and polygenic profile scores for CAD in GS:SFHS including all SNP thresholds.

**Page 10:** Supplementary Table 7, on the full analyses split by age groups between cognitive traits and polygenic profile scores for CAD in GS:SFHS including all SNP thresholds

**Page 11:** Supplementary Table 8, on the full analyses between cognitive traits and polygenic profile scores for CAD in LBC1936 and LBC1921 including all SNP thresholds.

**Page 12:** Supplementary Table 9, on the single SNP associations between specific SNPs for CAD and cognitive ability traits in GS:SFHS.


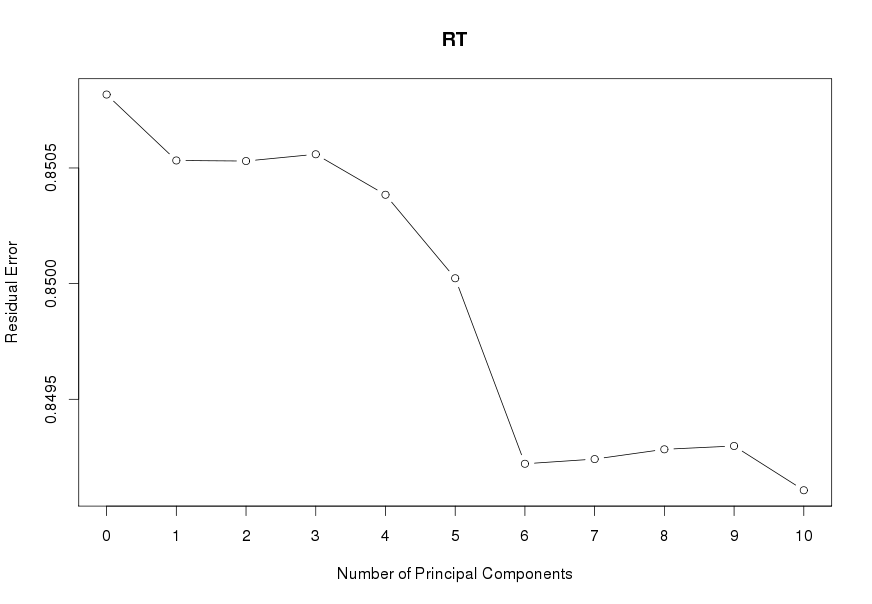
**Supplementary Figure 1a.** Comparison of log-Likelihoods for linear regression models of population stratification (up to 10 principal components)-adjusted cognition in GS:SFHS.


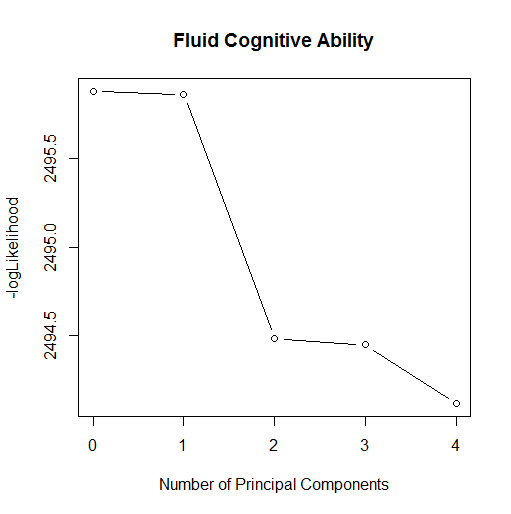


**Supplementary Figure 1b.** Comparison of log-Likelihoods for linear regression models of population stratification (up to 4 principal components)-adjusted cognition in LBC1936.

**
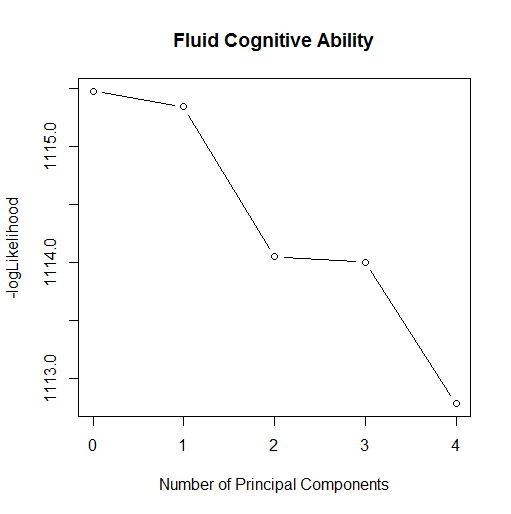
**

**Supplementary Figure 1c.** Comparison of log-Likelihoods for linear regression models of population stratification (up to 4 principal components)-adjusted cognition in LBC1921.

**Supplementary table 1.** Number of SNPs included at each threshold for the CAD polygenic risk scores at each threshold for the three cohorts.

| **Threshold** | **Number of SNPs GS:SFHS** | **Number of SNPs LBC1936** | **Number of SNPs LBC1921** |
| --- | --- | --- | --- |
| 1 | 115453 | 113117 | 112584 |
| 0.5 | 59365 | 58017 | 57797 |
| 0.1 | 12875 | 12589 | 12542 |
| 0.05 | 6819 | 6664 | 6642 |
| 0.01 | 1647 | 1629 | 1636 |

**Supplementary Table 2.** Proportion of variance explained in history of cardiovascular disease (diagnosed by a doctor) and polygenic risk for CAD derived using five different p-value thresholds in the UK Biobank sample.

| **Threshold** | **r^2^** | **p** |
| --- | --- | --- |
| 0.01 | 0.004407 | 1.72E-37 |
| 0.05 | 0.005754 | 2.19E-48 |
| 0.1 | 0.005605 | 3.68E-47 |
| 0.5 | 0.006323 | 6.03E-53 |
| 1 | 0.006182 | 8.1E-52 |

**Supplementary table 3a.** Phenotypic correlations for the GS:SFHS cognitive variables. All correlations p < 0.00001

| GS:SFHS | G_fluid_ | Verbal Intelligence | Memory | Verbal Fluency | Processing Speed |
| --- | --- | --- | --- | --- | --- |
| G fluid | - |  |  |  |  |
| Verbal Intelligence | 0.34 | - |  |  |  |
| Memory | 0.70 | 0.25 | - |  |  |
| Verbal Fluency | 0.66 | 0.39 | 0.17 | - |  |
| Processing Speed | 0.77 | 0.11 | 0.32 | 0.27 | - |

G_fluid_, fluid cognitive ability

**Supplementary table 3b.** Phenotypic correlations for the LBC1936 cognitive variables. All correlations p < 0.00001.

| LBC1936 | G_fluid_ | Verbal Intelligence | Memory | Processing Speed | Age 11 IQ | Age 70 IQ |
| --- | --- | --- | --- | --- | --- | --- |
| G fluid | - |  |  |  |  |  |
| Verbal Intelligence | 0.60 | - |  |  |  |  |
| Memory | 0.81 | 0.54 | - |  |  |  |
| Processing Speed | 0.75 | 0.43 | 0.55 | - |  |  |
| Age 11 IQ | 0.64 | 0.70 | 0.56 | 0.48 | - |  |
| Age 70 IQ | 0.69 | 0.69 | 0.60 | 0.52 | 0.71 | - |

G_fluid_, fluid cognitive ability

**Supplementary table 3c.** Phenotypic correlations for the LBC1921 cognitive variables. All correlations p < 0.00001.

| LBC1921 | G_fluid_ | Verbal Intelligence | Memory | Processing Speed | Age 11 IQ | Age 79 IQ |
| --- | --- | --- | --- | --- | --- | --- |
| G fluid | - |  |  |  |  |  |
| Verbal Intelligence | 0.64 | - |  |  |  |  |
| Memory | 0.65 | 0.32 | - |  |  |  |
| Processing Speed | 0.52 | 0.22 | 0.28 | - |  |  |
| Age 11 IQ | 0.63 | 0.64 | 0.29 | 0.24 | - |  |
| Age 79 IQ | 0.88 | 0.65 | 0.40 | 0.44 | 0.67 | - |

G_fluid_, fluid cognitive ability

**Supplementary table 4a.** Correlations between the five thresholds for the polygenic risk scores in GS:SFHS. All correlations p < 0.00001.

| **Threshold** | **1** | **0.5** | **0.1** | **0.05** | **0.01** |
| --- | --- | --- | --- | --- | --- |
| **1** | - |  |  |  |  |
| **0.5** | 0.98 | - |  |  |  |
| **0.1** | 0.75 | 0.76 | - |  |  |
| **0.05** | 0.63 | 0.65 | 0.85 | - |  |
| **0.01** | 0.41 | 0.42 | 0.57 | 0.67 | - |

**Supplementary table 4b.** Correlations between the five thresholds for the polygenic risk scores in LBC1936. All correlations p < 0.00001.

| **Threshold** | **1** | **0.5** | **0.1** | **0.05** | **0.01** |
| --- | --- | --- | --- | --- | --- |
| **1** | - |  |  |  |  |
| **0.5** | 0.98 | - |  |  |  |
| **0.1** | 0.74 | 0.77 | - |  |  |
| **0.05** | 0.65 | 0.67 | 0.86 | - |  |
| **0.01** | 0.41 | 0.43 | 0.59 | 0.69 | - |

**Supplementary table 4c.** Correlations between the five thresholds for the polygenic risk scores in LBC1921. All correlations p < 0.00001.

| **Threshold** | **1** | **0.5** | **0.1** | **0.05** | **0.01** |
| --- | --- | --- | --- | --- | --- |
| **1** | - |  |  |  |  |
| **0.5** | 0.98 | - |  |  |  |
| **0.1** | 0.77 | 0.78 | - |  |  |
| **0.05** | 0.67 | 0.68 | 0.86 | - |  |
| **0.01** | 0.48 | 0.47 | 0.6 | 0.7 | - |

**Supplementary Table 5.** Between-study heterogeneity results for GS:SFHS, LBC1921 and LBC1936

| CAD  genetic risk  scores:  SNPs with  P values < |  | **G_fluid_** | **VI** | **Memory** | **PS** | **CVD** |
| --- | --- | --- | --- | --- | --- | --- |
|  | | n = 11 121 | n = 11 121 | n = 11 225 | n = 10 985 | n = 7930 |
| **1** | I^2^ (%) | 21.8 | 25.1 | 57.8 | 25.3 | 13.6 |
|  | Q | 2.56 | 2.67 | 4.74 | 2.68 | 2.31 |
|  | p-value | 0.2784 | 0.263 | 0.0937 | 0.2622 | 0.3143 |
| **0.5** | I^2^ (%) | 8.6 | 32.3 | 57.3 | 50.9 | 0 |
|  | Q | 2.19 | 2.96 | 4.69 | 4.08 | 1.05 |
|  | p-value | 0.3347 | 0.2282 | 0.096 | 0.1302 | 0.5907 |
| **0.1** | I^2^ (%) | 60.7 | 21.5 | 71.5 | 79 | 49.3 |
|  | Q | 5.08 | 2.55 | 7.01 | 9.53 | 3.95 |
|  | p-value | 0.0787 | 0.2797 | 0.03 | 0.0085 | 0.1391 |
| **0.05** | I^2^ (%) | 13.5 | 49.9 | 31.1 | 81.9 | 0 |
|  | Q | 2.31 | 3.99 | 2.9 | 11.06 | 1.65 |
|  | p-value | 0.3149 | 0.1357 | 0.2343 | 0.004 | 0.4389 |
| **0.01** | I^2^ (%) | 0 | 12.9 | 0 | 67.9 | 71.7 |
|  | Q | 0.3 | 2.3 | 1.36 | 6.23 | 7.07 |
|  | p-value | 0.8603 | 0.3173 | 0.5075 | 0.0445 | 0.0292 |

G_fluid_, fluid cognitive ability; VI, verbal intelligence; PS, processing speed; CVD, history of self-reported cardiovascular disease.

**Supplementary Table 6.** Polygenic risk scores for CAD and cognitive abilities or self-reported history of cardiovascular disease in GS:SFHS, using mixed linear models implemented in ASReml-R.

| CAD polygenic risk score:  SNPs | **VI**  **(N=9697)**  **β, p** | **Memory**  **(N=9748)**  **β, p** | **VF**  **(N=9753)**  **β, p** | **PS**  **(N=9732)**  **β, p** | **G_fluid_**  **(N=9630)**  **β, p** | **CVD**  **(N=6413)**  **β, p** |
| --- | --- | --- | --- | --- | --- | --- |
| p<1 | -0.029, **0.0041**^†^ | -0.022, **0.0249** | -0.019, 0.0646 | -0.009, 0.3261 | -0.023, **0.0172**^†^ | 0.219, **0.0005** |
| p<0.5 | -0.029, **0.0035**^†^ | -0.021, **0.0346** | -0.020, 0.0534 | -0.008, 0.3591 | -0.023, **0.0199**^†^ | 0.202, **0.0013** |
| p<0.1 | -0.020, **0.0496** | -0.016, 0.1124 | 0.001, 0.8807 | -0.006, 0.5022 | -0.010, 0.3255 | 0.271, **1.40×^-5^** |
| p<0.05 | -0.020, **0.0458** | -0.013, 0.2046 | 0.008, 0.4208 | -0.001, 0.9575 | 0.002, 0.8487 | 0.202, **0.0011** |
| p<0.01 | -0.014, 0.1517 | -0.018, 0.0775 | 0.007, 0.4785 | 0.003, 0.7127 | 0.002, 0.8611 | 0.180, **0.0038** |

Effects with p-values < 0.05 are shown in bold. VI, verbal intelligence; VF, Verbal Fluency ; PS, processing speed; G_fluid_, fluid cognitive ability; CVD, history of self-reported cardiovascular disease. β, standardized regression coefficient; *p-*value derived from Wald Conditional F-test; †, association in subset excluding individuals with self-reported history of cardiovascular disease.

**Supplementary Table 7.** Polygenic Risk Scores for CAD and Cognitive Abilities for three different age groups in Generation Scotland at five SNP inclusion thresholds, using mixed linear models implemented in ASReml-R.

|  | | **< 40 years** | | | | |
| --- | --- | --- | --- | --- | --- | --- |
|  |  | **1** | **0.5** | **0.1** | **0.05** | **0.01** |
| **VI** | Beta | -0.024 | -0.025 | -0.017 | -0.019 | -0.022 |
| (N = 1804 ) | *p*-value | 0.236 | 0.211 | 0.383 | 0.330 | 0.262 |
| **Memory** | Beta | -0.014 | -0.016 | -0.012 | -0.015 | -0.004 |
| (N = 1810) | *p*-value | 0.511 | 0.458 | 0.588 | 0.495 | 0.860 |
| **VF** | Beta | -0.011 | -0.013 | 0.023 | 0.022 | 0.007 |
| (N = 1807) | *p*-value | 0.582 | 0.548 | 0.275 | 0.303 | 0.749 |
| **PS** | Beta | -0.014 | -0.016 | -0.020 | -0.018 | 0.003 |
| (N = 1818) | *p*-value | 0.481 | 0.445 | 0.336 | 0.393 | 0.896 |
| **G_fluid_** | Beta | -0.021 | -0.023 | -0.006 | -0.007 | -0.002 |
| (N = 1801) | *p*-value | 0.303 | 0.263 | 0.756 | 0.724 | 0.941 |
|  |  | **40-60    years** | | | | |
|  |  | **1** | **0.5** | **0.1** | **0.05** | **0.01** |
| **VI** | Beta | -0.021 | -0.023 | -0.009 | -0.007 | 0.007 |
| (N = 5144) | *p*-value | 0.115 | 0.084 | 0.504 | 0.596 | 0.519 |
| **Memory** | Beta | -0.025 | -0.025 | -0.016 | -0.012 | -0.017 |
| (N = 5158) | *p*-value | 0.057 | 0.057 | 0.231 | 0.371 | 0.200 |
| **VF** | Beta | 0.004 | 0.003 | 0.032 | 0.036 | 0.021 |
| (N = 5160) | *p*-value | 0.782 | 0.826 | **0.025** | **0.010** | 0.130 |
| **PS** | Beta | -0.004 | -0.005 | -0.001 | 0.006 | 0.007 |
| (N = 5165) | *p*-value | 0.750 | 0.647 | 0.982 | 0.618 | 0.570 |
| **G_fluid_** | Beta | -0.011 | -0.012 | 0.007 | 0.014 | 0.008 |
| (N = 5115) | *p*-value | 0.406 | 0.372 | 0.568 | 0.265 | 0.546 |
|  |  | **> 60 years** | | | | |
|  |  | **1** | **0.5** | **0.1** | **0.05** | **0.01** |
| **MHV** | Beta | -0.043 | -0.040 | -0.030 | -0.030 | -0.039 |
| (N = 2744) | *p*-value | **0.038** | 0.051 | 0.148 | 0.149 | 0.057 |
| **Memory** | Beta | -0.018 | -0.012 | -0.013 | -0.005 | -0.019 |
| (N = 2775) | *p*-value | 0.351 | 0.557 | 0.498 | 0.783 | 0.338 |
| **VF** | Beta | -0.065 | -0.063 | -0.052 | -0.033 | -0.007 |
| (N = 2781) | *p*-value | **0.001** | **0.001** | **0.008** | 0.093 | 0.729 |
| **PS** | Beta | -0.015 | -0.008 | -0.001 | 0.011 | 0.006 |
| (N = 2744) | *p*-value | 0.352 | 0.624 | 0.950 | 0.506 | 0.687 |
| **G_fluid_** | Beta | -0.044 | -0.037 | -0.027 | -0.010 | -0.003 |
| (N = 2709) | *p*-value | **0.017** | **0.044** | 0.138 | 0.576 | 0.852 |

Effects with p-values < 0.05 are shown in bold. VI, verbal intelligence; VF, Verbal Fluency; PS, processing speed; G_fluid_, fluid cognitive ability; Beta, standardized regression coefficient; *p-*value derived from Wald Conditional F-test

**Supplementary Table 8**. Polygenic risk scores for CAD and cognitive abilities or self-reported history of cardiovascular disease in LBC1921 & LBC1936

| CAD polygenic risk score:  SNPs | **IQ 11**  **β, p** | **IQ 79/70**  **β, p** | **G_fluid_**  **β, p** | **VI**  **β, p** | **Memory**  **β, p** | **PS**  **β, p** | **CVD**  **β, p** |
| --- | --- | --- | --- | --- | --- | --- | --- |
| **LBC1921** | (N=464) | (N=509) | (N=505) | (N=515) | (N=515) | (N=298) | (N=512) |
| P<1 | -0.018, 0.7039 | 0.024, 0.5846 | 0.044, 0.3270 | 0.041, 0.3533 | 0.069, 0.1200 | 0.084, 0.1357 | 0.221, **0.0085** |
| p<0.5 | -0.010, 0.8323 | 0.020, 0.6582 | 0.040, 0.3670 | 0.043, 0.3284 | 0.072, 0.1040 | 0.106, 0.0602 | 0.231, **0.0225** |
| p<0.1 | 0.003, 0.9607 | 0.066, 0.1387 | 0.086, 0.0558 | 0.052, 0.2368 | 0.102, **0.0212** | 0.172, **0.0033** | 0.184, 0.0692 |
| p<0.05 | 0.009, 0.8496 | 0.070, 0.1159 | 0.068, 0.1306 | 0.062, 0.1652 | 0.061, 0.1690 | 0.172, **0.0034** | 0.110, 0.2730 |
| p<0.01 | -0.003, 0.9506 | 0.049, 0.2703 | 0.023, 0.6035 | 0.050, 0.2588 | 0.012, 0.7840 | 0.146, **0.0131** | 0.166, 0.0975 |
| **LBC1936** | (N=944) | (N=991) | (N=986) | (N=1000) | (N=962) | (N=955) | (N=1005) |
| P<1 | -0.029, 0.376 | 0.006, 0.857 | -0.040, 0.194 | -0.006, 0.842 | -0.045, 0.150 | -0.004, 0.902 | 0.087, 0.239 |
| p<0.5 | -0.027, 0.404 | 0.007, 0.838 | -0.036, 0.238 | -0.004, 0.896 | -0.039, 0.215 | -0.001, 0.987 | 0.108, 0.142 |
| p<0.1 | -0.044, 0.182 | 0.001, 0.984 | -0.033, 0.285 | -0.014, 0.645 | -0.025, 0.427 | 0.014, 0.648 | 0.060, 0.418 |
| p<0.05 | -0.001, 0.965 | 0.032, 0.324 | 0.000, 0.992 | 0.013, 0.689 | 0.007, 0.813 | 0.055, 0.085 | 0.060, 0.418 |
| p<0.01 | -0.003, 0.924 | 0.021, 0.510 | -0.001, 0.936 | -0.028, 0.365 | 0.015, 0.634 | 0.025, 0.428 | -0.052, 0.482 |

Effects with p-values < 0.05 are shown in bold. IQ 11, IQ at age 11; IQ 79/70, IQ at age 79 (LBC1921) or 70(:BC1936); G_fluid_, fluid cognitive ability;

VI, verbal intelligence; PS, processing speed; CVD, history of self-reported cardiovascular disease; β, standardized regression coefficient.

**Supplementary Table 9.** Single SNP associations between specific SNPs for CAD (available in GS:SFHS) and the GS:SFHS cognitive ability traits (N = 6204).

| **SNP** | **VI**  **β, p** | **Memory**  **β, p** | **VF**  **β, p** | **PS**  **β, p** | **G_fluid_**  **β, p** |
| --- | --- | --- | --- | --- | --- |
| **rs11206510** | -0.011, 0.63 | 0.0038, 0.87 | 0.002, 0.94 | 0.015, 0.5154 | 0.010, 0.67 |
| **rs599839** | 0.005, 0.82 | 0.005, 0.81 | 0.029, 0.18 | 0.009, 0.67 | 0.020, 0.37 |
| **rs4845625** | -0.008, 0.66 | -0.003, 0.85 | -0.013, 0.48 | 0.0024, 0.89 | -0.006, 0.74 |
| **rs17464857** | 0.006, 0.80 | 0.018, 0.46 | -0.010, 0.68 | -0.002, 0.95 | 0.003, 0.90 |
| **rs2252641** | 0.029, 0.11 | -0.003, 0.85 | 0.014, 0.44 | -0.019, 0.28 | -0.005, 0.79 |
| **rs9818870** | 0.011, 0.66 | -0.014, 0.57 | 0.014, 0.56 | -0.046, 0.06 | -0.023, 0.34 |
| **rs9369640** | 0.040, 0.03 | 0.009, 0.65 | -0.012, 0.52 | -0.026, 0.16 | -0.015, 0.44 |
| **rs2048327** | 0.020, 0.28 | 0.030, 0.10 | 0.012, 0.52 | 0.046, 0.01 | 0.042, 0.02 |
| **rs4252120** | -0.022, 0.28 | -0.008, 0.70 | -0.004, 0.83 | -0.022, 0.26 | -0.017, 0.40 |
| **rs11556924** | -0.030, 0.10 | 0.002, 0.90 | -0.020, 0.27 | 0.001, 0.95 | -0.007, 0.68 |
| **rs3217992** | 0.027, 0.15 | 0.037, 0.05 | 0.006, 0.73 | -0.007, 0.70 | 0.016, 0.39 |
| **rs579459** | -0.008, 0.73 | 0.037, 0.11 | -0.005, 0.85 | 0.023, 0.32 | 0.027, 0.26 |
| **rs2047009** | 0.007, 0.71 | 0.020, 0.26 | 0.007, 0.72 | -0.006, 0.72 | 0.009, 0.62 |
| **rs501120** | 0.027, 0.32 | 0.018, 0.50 | 0.013, 0.63 | 0.025, 0.35 | 0.027, 0.32 |
| **rs11203042** | 0.007, 0.67 | 0.005, 0.78 | 0.003, 0.88 | 0.015, 0.40 | 0.011, 0.54 |
| **rs1412444** | -0.011, 0.54 | 0.002, 0.90 | -0.011, 0.54 | 0.003, 0.88 | -0.003, 0.88 |
| **rs974819** | -0.006, 0.76 | 0.020, 0.30 | 0.0012, 0.95 | 0.017, 0.36 | 0.019, 0.34 |
| **rs3184504** | -0.002, 0.89 | 0.037, 0.04 | 0.031, 0.08 | 0.023, 0.18 | 0.043, 0.017 |
| **rs4773144** | 0.00001, 0.99 | 0.033, 0.07 | -0.010, 0.60 | 0.020, 0.28 | 0.021, 0.26 |
| **rs9515203** | -0.017, 0.41 | -0.004, 0.86 | 0.021, 0.32 | 0.038, 0.06 | 0.027, 0.20 |
| **rs7173743** | 0.019, 0.30 | 0.024, 0.19 | 0.026, 0.15 | 0.025, 0.16 | 0.035, 0.05 |
| **rs17514846** | 0.025, 0.16 | 0.004, 0.83 | -0.008, 0.66 | 0.015, 0.38 | 0.006, 0.74 |
| **rs15563** | -0.007, 0.70 | 0.030, 0.09 | -0.010, 0.60 | 0.010, 0.58 | 0.015, 0.42 |
| **rs2075650** | 0.041, 0.10 | -0.050, 0.05 | 0.049, 0.05 | -0.018, 0.47 | -0.010, 0.68 |
| **rs445925** | -0.027, 0.36 | -0.030, 0.30 | -0.010, 0.74 | 0.012, 0.68 | -0.012, 0.68 |
